# Reply to the Comment Re: Magalhães-Sant’Ana, M. *Animals* 2019, *9*, 168

**DOI:** 10.3390/ani10071197

**Published:** 2020-07-15

**Authors:** Manuel Magalhães-Sant’Ana

**Affiliations:** 1 CIISA—Centre for Interdisciplinary Research in Animal Health, Faculty of Veterinary Medicine, University of Lisbon, 1300-477 Lisboa, Portugal; mdsantana@gmail.com; 2 Ordem dos Médicos Veterinários, Av. Filipe Folque, 10J, 4º Dto., 1050-113 Lisboa, Portugal

I am pleased that my viewpoint on traditional Chinese veterinary acupuncture (TCVA) has caught the attention of researchers from the China Agricultural University. In that paper [1], I rely on epistemological arguments to examine the conceptual, historical and scientific evidence regarding TCVA. Yusheng Hu and Zhongjie Liu (Hu & Liu) challenge some of my historical claims, namely the similarity between the nine-needle model and bloodletting instruments, the antiquity of veterinary acupuncture and some of the translations from the *Yuan Heng Liao Ma Ji* (*YHLMJ*). I find Hu & Liu’s letter very informative and useful to further clarify my views.

Hu & Liu point to the fact that I have based my claim on the similarities between the nine needles and European surgical instruments (namely lancets) on ‘only one illustration’, taken from the *Zhen Jiu Da Cheng*. That is, however, not just one illustration; it is the illustration chosen by the People’s Republic of China to promote traditional Chinese acupuncture in the West in the 1970s [2]. Moreover, Hu & Liu describe the names and functions of each of the nine needles. By doing so, Hu & Liu seem to fall in the same epistemological fallacy of their acupuncturist colleagues when describing the effects of acupuncture [1]. In order to defend that some needles are *not* lancets, Hu & Liu admit that others are, and that they were used for bloodletting. It is important to clarify that I use the term ‘lancet’ to designate any instrument used for lancing or puncturing blood vessels, abscesses or body cavities. If anything, the modern nine-needle model presented by Hu & Liu (Figure 1D) only made it clearer to the reader that the nine needles have little in common with contemporary filiform acupuncture needles. Even the thinnest needles in Figure 1D (numbers 2 and 3) are so long and thick that they would be better described as probes than needles. Finally, Hu & Liu present no evidence to support the claim that filiform needles existed since the Han Dynasty, other than the tautological argument that the practice of needling has been described in the *Huang Di Nei Jing*. However, the needling in ancient Chinese texts refers to the puncturing of vessels and abscesses or to the cauterization of wounds, and not to acupuncture [3].

The two remaining comments—the antiquity of veterinary acupuncture and the translations from the *YHLMJ*—will be dealt with together because they share the same rationale. Hu & Liu present substantial literature to claim that veterinary acupuncture is a thousand-year-old tradition. They also rely on contemporary translations of ancient Chinese texts to assert that acupuncture is profusely mentioned in the *Si Mu An Ji Ji* and the *YHLMJ* (cf. Supplementary material). In reality, these translations do not reflect the original meaning of those texts and are a clear case of how history has been re-interpreted to accommodate the dominant narrative of the antiquity of veterinary acupuncture. For example, the points in the illustration of a horse from one of the chapters of the *YHLMJ* (Figure 2) lack any reference to the theoretical system found in human acupuncture charts [4]. The misinterpretation of the word *zhen* for needling and the word *xue* for acupuncture points only adds to the confusion [4,5].

There is often little agreement on the exact translation of ancient Chinese symbols and translating the *YHLMJ* (Yuan Dynasty) was outside the scope of my article. This issue can only be satisfactorily resolved through the translation of original Chinese texts by independent scholars, an enterprise that I hope will be taken up by others. Either way, we all agree that the *YHLMJ* horse illustration in Figure 2a [1] alludes to the use of hair whorls in physiognomic divination and does not concern acupuncture. Even if its precise meaning remains elusive, it was chosen to highlight the similarities between the Chinese and European approaches to horse medicine, thus suggesting that they share a common origin [5].

Significantly, Hu & Liu’s comments are circumscribed to historical evidence and no attempt was made to challenge my conceptual claims (that the concepts employed by TCVA resemble those of humoral medicine and bloodletting) or my scientific claims that acupuncture is ineffective and works as a placebo. Nonetheless, Hu & Liu seem to recognize that the evidence on the clinical effects of acupuncture in companion animals is feeble. It should be noted, however, that the main reason why I focused on dogs rather than horses was because there is more evidence available and of better quality. My selection of case studies followed two criteria: the range of therapeutic claims (epilepsy, anesthesia, gut mobility, analgesia, rehabilitation medicine), and the quality of the journal in which they were published (*J. Small Anim. Pract.*, *J.A.V.M.A.*, *Acta Vet. Scand.*, *Res. Vet. Sci.*, *Vet. Rec.*, *PLoS ONE*). Judging by the SCIMAGO journal rankings, the presented studies are arguably amongst the best available, and yet provide no reliable evidence for the efficacy of TCVA.
